# The Contribution of Astrocyte Autophagy to Systemic Metabolism

**DOI:** 10.3390/ijms21072479

**Published:** 2020-04-03

**Authors:** Ana Ortiz-Rodriguez, Maria-Angeles Arevalo

**Affiliations:** 1 Instituto Cajal, Consejo Superior de Investigaciones Científicas (CSIC), 28002 Madrid, Spain; ana.ortiz@cajal.csic.es; 2 Centro de Investigación Biomédica en Red de Fragilidad y Envejecimiento Saludable (CIBERFES), Instituto de Salud Carlos III, 28029 Madrid, Spain

**Keywords:** astrocytes, autophagy, hypothalamus, metabolism, obesity

## Abstract

Autophagy is an essential mechanism to maintain cellular homeostasis. Besides its role in controlling the quality of cytoplasmic components, it participates in nutrient obtaining and lipid mobilization under stressful conditions. Furthermore, autophagy is involved in the regulation of systemic metabolism as its blockade in hypothalamic neurons can affect the central regulation of metabolism and impact body energy balance. Moreover, hypothalamic autophagy can be altered during obesity, one of the main alterations of metabolism nowadays. In this review, we focus on the role of astrocytes, essential cells for brain homeostasis, which represent key metabolic regulators. Astrocytes can sense metabolic signals in the hypothalamus and modulate systemic functions as glucose homeostasis and feeding response. Moreover, the response of astrocytes to obesity has been widely studied. Astrocytes are important mediators of brain inflammation and can be affected by increased levels of saturated fatty acids associated with obesity. Although autophagy plays important roles for astrocyte homeostasis and functioning, the contribution of astrocyte autophagy to systemic metabolism has not been analyzed yet. Furthermore, how obesity can impact astrocyte autophagy is poorly understood. More studies are needed in order to understand the contribution of astrocyte autophagy to metabolism.

## 1. Central Regulation of Metabolism

The central nervous system (CNS) plays an essential role in the control of body metabolism due to its capacity to detect peripheral signals that inform about energy status. Among these signals, nutrient availability (for instance levels of free fatty acids), and the hormones leptin and insulin are the major players. Leptin and insulin represent adiposity signals as their circulating levels are directly proportional to the amount of stored fat in the organism [1]. When nutrient availability and/or the levels of leptin and insulin are high, the brain inhibits caloric intake and glucose production. At the same time, energy expenditure and fat stores are increased. On the contrary, reduced levels of these circulating signals promote increased food intake and reduced energy expenditure in order to re-establish the energy balance [2]. 

The hypothalamus is one of the most important brain regions that govern feeding and energy expenditure. Among the diverse hypothalamic nuclei, the arcuate nucleus represents a key regulator of metabolic control due to its location. The arcuate nucleus is located on the ventral floor of the mediobasal hypothalamus and near the median eminence, a region rich in fenestrated capillaries, which allow passive diffusion of metabolites from the blood. This strategic location facilitates the transport of circulating hormones and nutrients, and their detection by the neurons of the arcuate nucleus. As a consequence, the arcuate nucleus can integrate peripheral metabolic signals and neuronal inputs, generating a coordinated response that allows for the regulation of body metabolism [3,4]. 

In the arcuate nucleus, two neuronal populations with antagonistic functions can be distinguished: NPY/AgRP neurons and POMC neurons. The first group co-expresses the orexigenic peptides neuropeptide Y (NPY) and agouti-related protein (AgRP). Fasting favors the activation of this neuronal population, promoting feeding and reducing energy expenditure. POMC neurons express proopiomelanocortin (POMC), the precursor from which α melanocyte-stimulating hormone (α-MSH) is synthetized. Both peptides have inhibitory effects on appetite (anorexigenic). POMC neurons are activated after feeding, favoring satiety feeling and promoting energy expenditure [4,5]. 

Despite the importance of the arcuate nucleus in the central control of metabolism, other hypothalamic regions as well as several brainstem nuclei and components of mesolimbic system participate in it. The signals coming from all of these regions are finally integrated with inputs from the decision-making centers, achieving a coordinated response for feeding, glucose metabolism, and energy expenditure [3,5]. 

## 2. Astrocyte Functions and Their Importance in Systemic Metabolism

Astrocytes are one of the most abundant cell types in the CNS. They were initially considered only physical and metabolic supporters of neurons. Nowadays, they have been involved in a wide range of functions, becoming essential for brain homeostasis and activity (Table 1).

Astrocytes participate in the formation and maintenance of the BBB. They are closely associated with brain capillaries through specialized processes, known as perivascular endfeet, which reach brain microvessels [6]. Through their perivascular endfeet, astrocytes can sense metabolic and endocrine signals from the blood. Simultaneously, astrocytic processes associate with neurons, mediating the transport of nutrients and establishing a close metabolic cooperation with this cell type [7,8]. Furthermore, astrocytes can regulate blood flow in response to changes in neuronal activity, a phenomenon known as neurovascular coupling [9]. Moreover, astrocytes are the brain cell type that predominantly stores glycogen, an energy reserve that can help to sustain neuronal activity during adverse conditions [10,11].

Astrocytes control ion and water homeostasis in the CNS, an essential function for neural excitability and several signaling processes [12]. Furthermore, although astrocytes are not electrically excitable cells, they represent important regulators of synaptic activity. Astrocytic processes reach the synaptic cleft, where they modulate ion and neurotransmitter concentrations through the presence of specific ion channels and neurotransmitter receptors. Moreover, astrocytes can release gliotransmitters in response to neuronal activity, modulating synaptic activity and strength. The important participation of astrocytes in synapses was coined as tripartite synapse, a concept that has broadened to multipartite synapse due to the involvement of microglia in it [13,14,15].

Another important function of astrocytes is their antioxidant role in the CNS. Astrocytes express a wide variety of antioxidant enzymes and store high amounts of glutathione and ascorbic acid, important antioxidant molecules. In addition, they release glutathione precursors that can be used by neurons to produce their own glutathione. Through all these mechanisms, astrocytes collaborate in scavenging reactive oxygen species (ROS) produced by neurons during oxidative phosphorylation [16,17,18]. Together with their antioxidant function, astrocytes participate in the response against CNS injuries in order to repair brain tissue [19,20].

In addition to their essential roles for brain homeostasis, astrocytes take part in the control of body metabolism. Although hypothalamic neuronal circuits involved in this function are characterized in detail, the role of astrocytes has emerged during the last decades. Astrocytes contain glucose transporters GLUT-1 and GLUT-2, which allow the participation of this cell type in hypothalamic and systemic sensing of glucose [21,22,23]. Furthermore, communication between hypothalamic astrocytes through gap-junctions is also necessary for correct glucose sensing in this region [24]. Together with glucose regulation, astrocytes are the main lipid metabolizers in the brain as it has been shown in vitro. Cultured brain astrocytes present higher capacity to use lipids in their oxidative metabolism than other cell types from the brain [25]. Furthermore, cortical astrocytes can take up fatty acids and incorporate them to other types of lipids better than neurons in vitro [26]. Moreover, production of ketone bodies by astrocytes in the hypothalamus can impact food intake. Local inhibition of ketone bodies production in the hypothalamus affects the feeding response after fasting in mice that had consumed a high fat diet (HFD). As ketone bodies are only produced by astrocytes in the brain, this study shows that hypothalamic astrocytes can participate in the regulation of the feeding response through ketone bodies release [27]. In addition to their capacity to detect nutrients, hypothalamic astrocytes express receptors for hormones that regulate body metabolism such as leptin [28] and insulin [29]. The exposure of hypothalamic astrocytes to these hormones can modulate their function. For instance, leptin modifies glucose and glutamate uptake of hypothalamic astrocytes in vitro [30]. Furthermore, specific elimination of leptin and insulin receptors in astrocytes is associated with systemic repercussions. Leptin receptor deletion in astrocytes modifies hypothalamic neuronal circuits and food intake patterns [31]. When the insulin receptor is eliminated in this cell type, hypothalamic glucose sensing and systemic metabolism of glucose are impaired [29]. Therefore, astrocytes participate in important systemic functions such as food intake or glucose metabolism through hormonal signaling.

Finally, astrocytes play an essential role in the inflammatory response of the brain during obesity. Increased expression of glial fibrillary acidic protein (GFAP), an astrocyte marker, is seen in the hypothalamus after HFD consumption. Moreover, the morphology of astrocytes acquires a reactive phenotype [32]. These morphological changes observed in astrocytes are linked to alterations in the hypothalamic neuronal circuits. HFD intake increases the glial coverage of NPY and POMC neurons, decreasing the synaptic inputs received by these neurons [33]. The response of astrocytes to HFD consumption is extremely fast, as increased expression of proinflammatory cytokines and GFAP is observed only one day after. This early proinflammatory response is attenuated seven days after HFD onset, but it reappears if HFD intake is prolonged [32]. Recent studies have shown that this initial activation of astrocytes could be a protective mechanism to adapt to the new metabolic situation. Morphological changes are described in astrocytes between fasting and fed status in mice [34]. Furthermore, inactivation of the inflammatory response of astrocytes before HFD consumption increases food intake of mice [35]. On the contrary, blockade of inflammatory signaling in astrocytes after HFD onset protects mice from metabolic alterations [36]. Therefore, initial activation of astrocytes could play a protective role, while its chronic activation is responsible for metabolic alterations associated with HFD.

## 3. Autophagy Importance in Metabolism

Autophagy is a catabolic process that allows the degradation of cytoplasmic material through lysosomal action in mammals. Three different types of autophagy are distinguished by the mechanism used to deliver cytoplasmic components to lysosomes: macroautophagy, chaperone-mediated autophagy, and microautophagy. Macroautophagy (autophagy hereafter) is characterized by the sequestration of cytoplasmic components into double-membrane vesicles, known as autophagosomes, which finally fuse with lysosomes. In chaperone-mediated autophagy, proteins that contain a specific sequence of amino acids (KFERQ motif) are directly targeted to lysosomes. The KFERQ motif allows protein recognition by cytoplasmic chaperones that interact with lysosome-associated membrane protein 2A (LAMP-2A). Due to this interaction, targeted proteins can be translocated into the lysosomal lumen for degradation. Finally, microautophagy consists of direct sequestration of cytoplasmic material by lysosomes through invagination of lysosomal membrane. In mammals, microautophagy is carried out by late endosomes instead of lysosomes [37,38,39].

Autophagy is constitutively active at low levels and acts as a mechanism of quality control for cytoplasmic components. Through basal autophagy, proteins and organelles that are unnecessary or damaged can be eliminated. Furthermore, autophagy is induced in the presence of several stressors such as nutrient deprivation, oxidative stress, pathogen infection, and hypoxia. Under these conditions, autophagy enables metabolic adaptation and promotes cell survival. Among the stressors above-mentioned, nutrient deprivation is the most potent activator of autophagy in cells. During starvation, autophagy allows for the production of amino acids that can be used as an energy source and to synthetize essential proteins for cell survival [40,41,42]. Together with amino acid supply during starvation, autophagy can provide free fatty acids. This process, known as lipophagy, mobilizes lipids from lipid droplets through autophagic machinery. Deletion of essential genes for autophagy (*Atg5* and *Atg7*) causes lipid accumulation in hepatocytes due to reduced lipolysis in vitro and in vivo [43,44]. Lipophagy has also been described in fibroblasts, macrophage foam cells, T cells, neurons, and glial cells [43,45,46,47], suggesting its importance as a general mechanism to mobilize lipids independently of cell type.

Apart from autophagy importance in cell metabolism, several studies have shown its participation in the central regulation of metabolism and energy balance. Autophagy is active in the arcuate nucleus in basal conditions [48,49] and plays important functions on AgRP and POMC neurons. In AgRP neurons, autophagy allows the mobilization of neuronal lipids and production of AgRP peptide under starvation conditions. When autophagy is inhibited in AgRP neurons, AgRP production in response to fasting is blunted. Furthermore, POMC and α-MSH levels are increased independently of the nutritional status of mice, which contributes to the reduced fat mass and body weight of these animals [50]. In comparison with the previous study, inhibition of autophagy in POMC neurons increases adiposity and body weight [49,51,52]. These changes in phenotype are associated with a reduced production of α-MSH and lipid mobilization in adipose tissue after fasting [51]. Furthermore, glucose regulation and leptin sensitivity are altered [49,51,52]. Moreover, axonal projections of POMC neurons to other hypothalamic nuclei are diminished, showing the importance of autophagy for the normal development of this neuronal population [49]. These studies manifest the relevance of autophagy for the correct functioning of AgRP and POMC neurons, essential players in the central control of metabolism. In addition, hypothalamic autophagy can control lipophagy in the periphery. Stimulation of hypothalamic autophagy promotes lipid mobilization in brown adipose tissue and liver through lipophagy [53].

In relation to obesity, autophagy alterations have been described in the hypothalamus. Prolonged consumption of HFD downregulates the levels of autophagy markers in the hypothalamus of rodents [48,54,55]. Furthermore, an accumulation of the autophagy substrate p62 and ubiquitin is described, suggesting a blockade of autophagy in the hypothalamus after HFD consumption [54,55]. Together with a reduction in general activity of autophagy, obesity can affect selective forms of autophagy. Reduced autophagy of mitochondria and lipids is described in the hypothalamus after HFD intake [56]. Furthermore, additional blockade of autophagy by *Atg7* deletion in POMC neurons exacerbates HFD impact on mice, increasing body weight and impairing glucose homeostasis under these conditions [51,52]. Finally, maternal obesity can impact hypothalamic autophagy in the offspring. HFD administration during pregnancy reduces the incorporation of microtubule-associated protein 1A/1B light chain 3B (LC3) to autophagosomes and accumulates p62 in the offspring hypothalamus at weaning. Moreover, this early exposure to HFD affects hypothalamic autophagy in response to HFD re-exposure during adulthood [57].

## 4. Autophagy in Astrocytes

As previously mentioned, adaptation to starvation is the most conserved function of autophagy. In the case of astrocytes, autophagy represents an essential mechanism to face the lack of nutrients. Several studies have shown that amino acid deprivation or ATP depletion activates autophagy in cultured astrocytes [58,59]. The blockade of autophagy using chloroquine (a drug that impairs autophagosome fusion with lysosomes) exacerbates astrocyte cell death after nutrient deprivation [60]. These studies reveal that autophagy is activated during starvation to promote cell survival in astrocytes.

Apart from its role during starvation, autophagy represents a quality control mechanism to avoid protein aggregation. Accumulation of cytoplasmic protein inclusions is a common feature of neurodegenerative diseases. Astrocytes, which contribute to the development of these disorders, also show protein inclusions in their cytoplasm [61]. Initial studies described that autophagy deficits in CNS caused an accumulation of protein aggregates only in neurons, inducing neurodegeneration in mice [62,63]. However, astrocytes can modulate their autophagic response to prevent the formation of these inclusions. For instance, impairment of proteasome activity generates an accumulation of protein aggregates in the cytoplasm of astrocytes. Through autophagy activation, astrocytes achieve the reduction of protein accumulation and promote cell viability [64]. Autophagy is also modulated in astrocytes affected by Alexander’s disease, a disorder caused by mutations in the *GFAP* gene. Under these conditions, astrocytes activate their autophagy to degrade GFAP and avoid its accumulation [65]. Another neurodegenerative disease characterized by the presence of cytoplasmic inclusions is Parkinson’s disease. α-synuclein inclusions do not only accumulate in the cytoplasm of dopaminergic neurons, but also in astrocytes [66]. Some studies have shown that autophagy modulation in astrocytes can affect α-synuclein accumulation in the brain. When autophagy is inhibited by αB-crystallin (a small heat shock protein implicated in protein aggregation), the clearance of α-synuclein pre-formed fibrils is reduced in astrocytic cytoplasm. Furthermore, specific overexpression of αB-crystallin in astrocytes and its consequent inhibitory effect on autophagy generate a greater accumulation of α-synuclein in the brain of a Parkinson’s disease mouse model [67]. Familiar forms of Parkinson’s disease are linked to mutations in leucine-rich repeat kinase 2 (*LRRK2*), a gene involved in autophagy among several other functions [68]. Astrocytes derived from induced pluripotent stem cells of patients with mutations in *LRRK2* show α-synuclein accumulation in their cytoplasm. This accumulation is associated with impaired macroautophagy and chaperone-mediated autophagy, and can be prevented by using inducers of chaperone-mediated autophagy [69]. Together with the importance of autophagy for protein degradation in the cytoplasm of astrocytes, it participates in the elimination of extracellular amyloid plaques in Alzheimer’s disease. Astrocytes that carry the *ε*4 allele of apolipoprotein E (APOE), an allele associated with a higher risk of Alzheimer’s disease, have a reduced autophagic flux and impaired capacity to clear amyloid plaques in a mouse model of the disease. Moreover, induction of autophagy by rapamycin promotes Aβ plaques clearance, highlighting the role of autophagy in this astrocytic function [58]. All these studies manifest the relevance of astrocytic autophagy in the regulation of protein degradation and its important implications for neurodegeneration.

Autophagy is also involved in astrocyte differentiation during cortex development in mice. Atg5 knockdown reduces the differentiation of neural progenitor cells into astrocytes both in vitro and in vivo. On the contrary, an increased number of astrocytes is found when this protein is overexpressed, showing the importance of Atg5 in astrocyte differentiation [70]. Furthermore, autophagy is implicated in the differentiation of adult hippocampal neural stem cells into astrocytes. This differentiation process is associated with increased autophagic flux in vitro. Furthermore, genetic or pharmacological inhibition of autophagy affects astrocyte differentiation, reducing the number of GFAP-positive cells [71]. Finally, autophagy can affect astrocyte transformation into gliomas. Unc-51, like autophagy activating kinase 2 (*ULK2*), an inducer of autophagy, is hypermethylated and its expression is reduced in glioma. Ectopic expression of ULK2 reduces Ras-induced transformation of astrocytes, showing an inhibitory role of autophagy in glioma transformation [72].

Astrocytes can adapt their metabolic function to provide energy in the presence of CNS insults. During inflammatory response, changes in the metabolic profile of reactive astrocytes have been described [7,73]. Furthermore, a rearrangement of mitochondrial networks occurs in astrocytes under these circumstances. After being exposed to an inflammatory stimulus, astrocyte mitochondria become fragmented. However, this response is transient as mitochondria finally recover their tubular morphology. During mitochondrial rearrangement, autophagy is activated and fragmented mitochondria are engulfed by autophagosomes. Lack of autophagy avoids the recovery of tubular mitochondria, favoring ROS production, and affecting cell viability [74].

Together with its participation in mitochondrial remodeling, autophagy contributes to astrocyte activation and death after brain lesions such as cerebral ischemia and traumatic brain injury. Autophagy is induced in cortical astrocytes after permanent middle cerebral artery occlusion [75]. Furthermore, autophagy activation is also described in cultured astrocytes exposed to oxygen-glucose deprivation, a model which mimics ischemic conditions in vitro. Increased levels of Beclin-1, LC3-II/LC3-I ratio, and autophagic vesicles are found in astrocytes exposed to this type of deprivation. Inhibition of autophagy using 3-methyladenine (3-MA) or bafilomycin reduces autophagy activation and counteracts cell death [75]. Furthermore, astrocyte activation is reduced after autophagy inhibition by 3-MA in this in vitro model [76]. Autophagy activation is also described in astrocytes exposed to glutamate, an in vitro model that imitates glutamate excitotoxicity seen in traumatic brain injury. Induction of autophagy is accompanied by astrocyte death under these conditions. Autophagy inhibition by 3-MA reduces astrocyte death, showing that autophagy contributes to cytotoxicity caused by glutamate [77].

Viral infection can also modulate autophagy in astrocytes and one example of this is human immunodeficiency virus (HIV). Astrocytes are a target for HIV in the CNS, maintaining a latent infection and acting as a possible reservoir of this virus [78,79]. Astrocytes can modulate their autophagic activity as a protective mechanism to avoid HIV-induced cell death. Productive HIV infection promotes an activation of mitophagy in human astrocytes, favoring selective elimination of damaged mitochondria by autophagy. Through this mechanism, production of mitochondrial ROS is reduced and mitochondrial membrane potential is maintained, which promotes astrocyte survival [80]. Furthermore, the use of autophagy inducers (rapamycin) and inhibitors (3-MA and leupeptin) reduces or promotes human astrocyte cytotoxicity, respectively [81].

Several compounds with protective effects for the brain act through the modulation of autophagy in astrocytes. For instance, progesterone exerts anti-inflammatory effects on astrocytes exposed to β-amyloid through autophagy activation. When autophagy is inhibited by 3-MA, the protective effect of progesterone is lost and the transcription of proinflammatory cytokines is increased in astrocytes [82]. In the case of resveratrol, it can counteract autophagy alterations after glutamate exposure and promote astrocyte viability [77]. In addition, the antidepressant fluoxetine protects cultured astrocytes from the deleterious effect of corticosterone through the induction of autophagic flux. Fluoxetine concretely increases mitophagy, which allows the elimination of damaged mitochondria and alleviates ROS production induced by corticosterone in astrocytes [83].

Finally, astrocyte autophagy also affects neuronal survival. Autophagy blockade caused by lysosomal dysfunction in astrocytes induces degeneration of cortical neurons in vivo [84]. Furthermore, autophagy modulation in astrocytes can regulate neuronal viability after harmful stimuli. In a model of oxygen and glucose deprivation followed by reoxygenation, autophagy induction by rapamycin in astrocytes reduces neuronal death. In contrast, autophagy inhibition in astrocytes increases neuronal death in this in vitro model [85]. Furthermore, the specific upregulation of autophagy in astrocytes reduces infarct volume and neuronal loss in an in vivo model of middle cerebral artery occlusion with later reperfusion [85].

**Figure 1:**
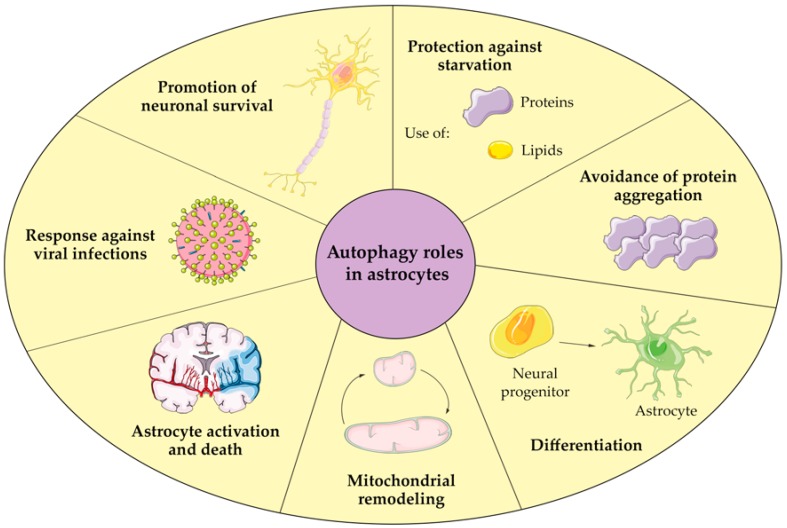
Autophagy functions in astrocytes.

All these studies show the involvement of autophagy in a wide variety of functions in astrocytes, ranging from their development to their homeostasis and survival (Figure 1). For this reason, autophagy represents an essential mechanism for the correct function of astrocytes. Furthermore, due to the essential role of astrocytes in maintaining brain homeostasis and functions, astrocyte autophagy can affect brain activity and deserves special attention.

## 5. Involvement of Astrocyte Autophagy in Systemic Metabolism

Although autophagy has been identified as an essential mechanism for the central regulation of metabolism, all of the studies have only explored the role of neuronal autophagy [48,49,51,52]. As has been described above, astrocytes are key players in metabolic control and their autophagy exerts important roles for brain functioning. However, the contribution of astrocyte autophagy to the regulation of systemic metabolism has not been established yet.

It is important to take into consideration that autophagy can be differentially regulated in neurons and astrocytes. For instance, activation of autophagy after starvation is more pronounced in cultured astrocytes than neurons [86]. Moreover, acute ethanol exposure enhances autophagy in cultured astrocytes. This induction of autophagy represents a protective mechanism that tries to avoid astrocyte inflammation and cell death under these conditions. However, ethanol downregulates autophagy in neuronal cultures, making neurons more sensitive to ethanol toxicity [87]. Furthermore, autophagy and lysosomal biogenesis could differ between glia and CA1 neurons in the brain of Alzheimer’s disease patients. This is due to increased nuclear translocation of transcription factor EB (TFEB), a master regulator of these processes, in glial cells compared to neurons [88]. The existence of all these differences shows the complexity of autophagy regulation in the brain and highlights the necessity of studying the contribution of autophagy to systemic metabolism in each cell type. Body metabolism should be characterized in animal models with specific autophagy inhibition in astrocytes. After deleting essential genes for autophagy in astrocytes, several metabolic parameters like body weight and composition, glucose homeostasis and leptin sensitivity should be measured in these animals. Together with this approach, it would be interesting to examine the role of astrocyte autophagy specifically in the hypothalamus. Through lentiviral injection in the hypothalamus, autophagy could be inhibited only in the astrocytes located in this region. Moreover, the morphology of astrocytes and the glial coverage of hypothalamic neurons could be analyzed in order to establish the impact of this inhibition on hypothalamic circuits. Finally, all the studies performed should include animal models in which different genes of the autophagy machinery are deleted. It is important to take into account that autophagy proteins can also mediate non-autophagic functions [89,90]. For this reason, we could only attribute the observed changes to autophagy if these alterations were shared by animals that carry deletions of different genes involved in autophagy.

Apart from determining how astrocyte autophagy can impact metabolic regulation, it would be interesting to determine its contribution to obesity pathophysiology. As we have described above, hypothalamic obesity is altered during obesity. In particular, these studies only collected data from the hypothalamus in general [48,54,55] or analyzed the effect of HFD after autophagy inhibition in POMC neurons [51,52]. However, the impact of HFD intake on autophagy has not been widely examined in astrocytes. The only available data are the description of increased LC3-II levels in hippocampal astrocytes after chronic HFD [91]. Higher LC3-II levels correlate with an increased number of autophagosomes in the cytoplasm [92]. Nevertheless, autophagy is a very dynamic process and an elevated number of autophagosomes can be associated with increased autophagosome formation or reduced degradation. For this reason, measuring only LC3-II levels is not enough to determine the status of autophagy in astrocytes after HFD consumption [93].

Together with hypothalamic inflammation, obesity is characterized by alterations in lipid content of the brain. HFD consumption causes an abnormal accumulation of saturated fatty acids like palmitic acid (PA) in the brain of mice [94,95]. Moreover, metabolic syndrome patients show higher incorporation of free fatty acids in the brain [96]. To study the impact of saturated fatty acids on the brain, an in vitro model consisting of treating the different brain cell types with PA has been developed. In the case of astrocytes, PA decreases glucose uptake and lactate release [97]. Furthermore, it increases the production of proinflammatory cytokines [94,98] and ROS in astrocytes [99,100]. As a consequence of these alterations, PA induces apoptosis and decreases cell viability of astrocytes [99,101].

Using this in vitro model, the impact of saturated fatty acids on astrocyte autophagy was analyzed. PA exposure can modulate autophagy in astrocytes, but autophagy blockade and induction has been described [91,102]. PA simultaneously increases LC3-II and p62 levels in cultured astrocytes [102]. The existence of high levels of LC3-II and accumulation of p62 suggested a reduction in autophagic activity. To confirm the blockade of autophagy, autophagic flux was measured using hydroxychloroquine, a lysosomal inhibitor that avoids autophagosome fusion with lysosomes. LC3-II accumulation caused by hydroxychloroquine was reduced after PA treatment, which confirms the blockade of autophagy in astrocytes [102]. A reduction in autophagic activity has also been described in hypothalamic neurons exposed to PA [103]. On the contrary, autophagy induction by PA has also been reported in cultured astrocytes [91]. In this study, increased levels of LC3-II and autophagosome accumulation were described in astrocytes after PA exposure. Although autophagy flux and levels of autophagic substrates were not quantified, the inhibition of autophagosome formation by 3-MA avoided the changes induced by PA [91]. These results suggest that autophagy is induced by PA in astrocytes, an opposite result compared to the blockade of autophagy found by Ortiz-Rodriguez et al. [102]. The differences between these two studies could be caused by the use of astrocytes from different brain regions, as the response of cortical [102] and hippocampal [91] astrocytes was analyzed. For instance, differences in the response to PA between hypothalamic and cortical astrocytes have been reported [104], which could explain the differences found in astrocyte autophagy.

As can be seen, few studies have explored how astrocyte autophagy can be modulated during obesity. Studying the metabolic response to HFD in animal models when astrocyte autophagy is inhibited could help us to understand the role of astrocyte autophagy under these conditions. Furthermore, contradictory results have been reported in the in vitro model of PA treatment. We encourage scientists in the field to develop new in vitro studies to figure out the molecular mechanisms that follow autophagy alterations and could contribute to astrocyte dysfunction after the exposure to saturated fatty acids. As can be seen, more studies are needed in order to understand the role of autophagy for astrocyte contribution to obesity. Furthermore, dissecting the implication of astrocyte autophagy in metabolic regulation and its alterations could help to find new therapeutic targets to develop new anti-obesity drugs.

## Figures and Tables

**Table 1:** Main functions of astrocytes in the brain.

Blood–brain barrier (BBB) formation and maintenance	[6]
Nutrient transport to neurons	[7,8]
Regulation of cerebral blood flow depending on neuronal activity	[9]
Glycogen storage	[10,11]
Control of ion and water homeostasis	[12]
Modulation of synaptic activity	[13,14,15]
Antioxidant defense	[16,17,18]
Response against CNS injuries	[19,20]

## Abbreviations

3-MA	3-methyladenine
α-MSH	α melanocyte-stimulating hormone
AgRP	Agouti-related protein
APOE	Apolipoprotein E
BBB	Blood–brain barrier
CNS	Central nervous system
GFAP	Glial fibrillary acidic protein
HFD	High fat diet
HIV	Human immunodeficiency virus
LAMP-2A	Lysosome-associated membrane protein 2A
LC3	Microtubule-associated protein 1A/1B light chain 3B
LRRK2	Leucine-rich repeat kinase 2
NPY	Neuropeptide Y
PA	Palmitic acid
POMC	Proopiomelanocortin
ROS	Reactive oxygen species
TFEB	Transcription factor EB
ULK2	Unc-51 like autophagy activating kinase 2
